# Potent anti-cancer effects of less polar Curcumin analogues on gastric adenocarcinoma and esophageal squamous cell carcinoma cells

**DOI:** 10.1038/s41598-017-02666-4

**Published:** 2017-05-31

**Authors:** Fatemeh Alibeiki, Naser Jafari, Maryam Karimi, Hadi Peeri Dogaheh

**Affiliations:** 1 School of Medicine, Ardabil University of Medical Science, Ardabil, 56197 Iran; 2 Markey Cancer Center, University of Kentucky, Lexington, KY USA; 3 Department of Medicinal Chemistry, Faculty of Pharmacy and Pharmaceutical Sciences Research Center, Tehran University of Medical Science, Tehran, 14176 Iran

## Abstract

Curcumin and its chalcone derivatives inhibit the growth of human cancer cells. It is reported that replacement of two OH groups in curcumin with less polar groups like methoxy increases its anti-proliferative activity. In this study, we explored benzylidine cyclohexanone derivatives with non-polar groups, to see if they possess increased anti-cancer activity. Novel 2,6-bis benzylidine cyclohexanone analogues of curcumin were synthesized, and their inhibitory effects on gastric adenocarcinoma (AGS) and esophageal squamous cell carcinoma (KYSE30) cancer cells were studied using an MTT assay. Cell apoptosis was detected by EB/AO staining, and cell cycle was analyzed by flow cytometry. Real-time PCR was performed for gene expression analysis. All synthesized analogues were cytotoxic toward gastric and esophageal cancer cells and showed lower IC_50_ values than curcumin. Treatment with 2,6-Bis-(3-methoxy-4-propoxy-benzylidene)-cyclohexanone (BM2) was 17 times more toxic than curcumin after 48 h incubation. All novel compounds were more effective than curcumin in apoptosis induction and cell cycle arrest at G1 phase. These results suggest that less polar analogues of curcumin have potent cytotoxicity *in vitro*. However, they need to be investigated further, especially with animal tumor models, to confirm their chemotherapeutic activity *in vivo*.

## Introduction

Curcumin is the most active component of rhizomes of *Curcuma longa L*. (also known as turmeric) and is classified in the family Zingiberaceae. It has been studied extensively due to its bio-functional properties, especially antioxidant, anticancer, anti-growth, antiarthritic, antiatherosclerotic, antidepressant, antiaging, antidiabetic, antimicrobial and anti-inflammatory activities^1^.

These activities have been attributed to methoxy, hydroxyl, α, β-unsaturated carbonyl moiety, or diketone groups in curcumin^2^.

Curcumin’s key mechanisms of chemotherapeutic action include inducing apoptosis; inhibiting proliferation, migration and invasion of tumors; and also weakening tumors to radiotherapy and chemotherapy. At a molecular level, this multi-targeted agent has been shown to exhibit anti-inflammatory activity through suppression of numerous cell signaling pathways including NF-κB, STAT3, NRF2, ROS, and COX2^3^.

Several studies reported curcumin’s anti-cancer effects on gastrointestinal cancers, leukemia, breast cancer, genitourinary cancers, ovarian cancer, head and neck squamous cell carcinoma, lung cancer, melanoma, neurological cancers, and sarcoma^4^, which demonstrate its potential capability to affect multiple targets.

Despite all these facts, the utility of curcumin is limited by its low bioavailability^4^. Thus, modifications of specific functional groups of curcumin that increase its bioavailability will increase its activity. Developing structural analogues of curcumin and synthesis of “man-made” curcumin analogues plays a role in the improvement of its bioavailability. For instance, the natural analogues of curcumin such as dimethoxy-curcumin and bidemethoxy-curcumin were reported to have similar biological activity to curcumin^5^. Moreover, it has been reported that chalcone and bis-chalcone derivatives have inhibited the growth of the human breast and colon cancer cell lines^6^.

In this study, our purpose was to explore efficacy of benzylidine cyclohexanone derivatives with methoxy, ethoxy, alkoxy, and propoxy groups, to see if they possess increased anti-cancer activity and to explore the mechanism of action of these analogues. In order to accomplish this, we developed a new series of 2,6-bis benzylidine cyclohexanone derivatives that indicate increased activity against gastric and esophageal cancer cells *in vitro*, compared to treatment with unmodified curcumin.

## Methods

### Synthesis of 2,6-bis benzylidine cyclohexanone derivatives

Benzaldehydes including 4-hydroxy benzaldehyde and 4-hydroxyl, 3-Methoxy benzaldehyde (5 mM) were dissolved in 25 mL solution of N,N-Dimethylformamide (DMF) including potassium carbonate (K_2_CO_3_, 6 mM) as a catalytic base for 1 h at 80 °C. Afterward, individual reactions received one of the propargyl bromide, allyl bromide, or iodoethane. Then mixtures were heated and mixed for 24 h.

In order to extract benzaldehydes from DMF, 100 mL water and 50 mL ethyl acetate were added to mixtures and organic phases were isolated. Then compounds were dried on sodium sulfate, and finally ethyl acetate was evaporated.

**Figure 1:**
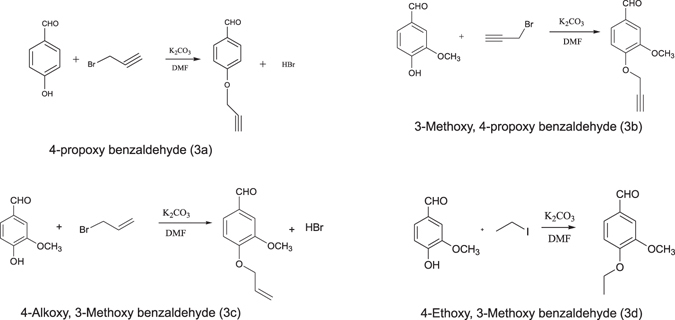
Synthesized primary aromatic aldehydes.

Thin-layer chromatography (TLC) was conducted to determine the purity of products. The selected aromatic aldehydes were as follows: 4-propoxy benzaldehyde (3a), 3-methoxy, 4-propoxy benzaldehyde (3b), 4-Alkoxy, 3-Methoxy benzaldehyde (3c), 4-ethoxy, and 3-methoxy benzaldehyde (3d) (Fig. 1).

**Figure 2:**
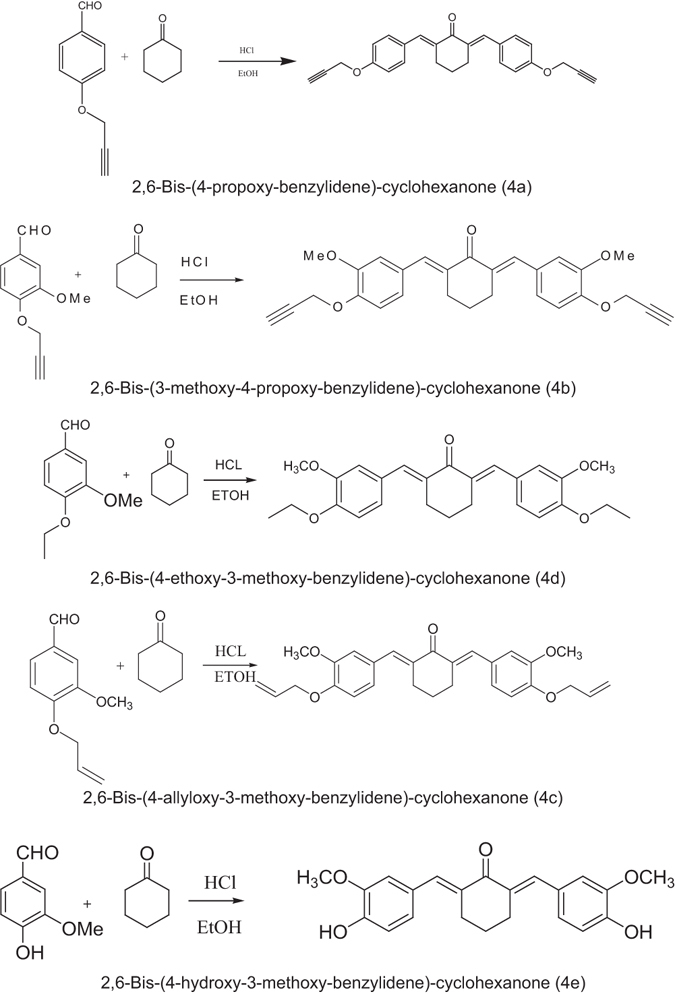
Synthesized 2,6-bis benzylidene cyclohexanones (4a – 4e also named BM1- BM5).

2,6-bis benzylidene cyclohexanones (4a – 4e) were prepared by reacting 2 equivalents of aromatic aldehydes with 1 equivalent of cyclohexanone in the presence of ethanol and hydrochloric acid gas. Then the compounds were washed with cold ethanol and verified by TLC (Fig. 2). Finally, products were characterized and analyzed by ^13^C-NMR, ^1^H-NMR and FT-IR.

Details of generated 2,6-bis benzylidene cyclohexanones including formula, structure, molecular weight, melting point, color and yield are presented in Table 1.

**Table 1:** Chemical characterization of the synthesized 2,6-bis benzylidene cyclohexanones including formula, structure, molecular weight, melting point, color and yield.

	Name	Formula	Structure	MW	Melting Point (**°C**)	Color	Yield
4a	2,6-Bis-(4-propoxy-benzylidene)-cyclohexanone (BM1)	C_26_H_22_O_3_		283.45	158–160.2	Yellow	78%
4b	2,6-Bis-(3-methoxy-4-propoxy-benzylidene)-cyclohexanone (BM2)	C_28_H_26_O_5_		442.50	162.9–164.2	Green	89%
4c	2,6-Bis-(4-allyloxy-3-methoxy-benzylidene)-cyclohexanone (BM3)	C_28_H_30_O_5_		446.53	182.9–184.3	Brown	85%
4d	2,6-Bis-(4-ethoxy-3-methoxy-benzylidene)-cyclohexanone (BM4)	C_26_H_30_O_5_		422.51	172.9–174.5	phosphorous	80%
4e	2,6-Bis-(4-hydroxy-3-methoxy-benzylidene)-cyclohexanone (BM5)	C_22_H_22_O_5_		366.41	145.5–147	Brown	87%

### Cell Culture and Treatment

The human gastric adenocarcinoma (AGS) and esophageal squamous cell carcinoma (KYSE30) cell lines were provided by the Pasteur Institute of Iran. All reagents, chemicals and media were prepared and used freshly.

Cancer cells were grown in RPMI-1640 medium, supplemented with 10% fetal bovine serum (FBS), penicillin (100 unit/ml) and streptomycin (100 μg/mL). Cells were cultured at 37 °C in a moistened atmosphere of 5% CO_2_ and 95% air. Then, cells were trypsinized and plated in 96-well plates at a density of 1 × 10^4^ cells per well in 150 μl medium, and incubated overnight. Next, cells were treated with a FBS-free medium containing 1 mg/ml of each compound by 1/4 serial dilutions. Then, plates were incubated for 24, 48, and 72 h. The cytotoxicity of curcumin derivatives was determined by an MTT assay.

### MTT Assay

Culture media were removed 4 h before completion of the incubation time, then 200 μl of 0.25 mg/ml MTT (Merck, Germany) was added to each well. Plates were incubated again for an additional 4 h in order to complete the incubation time. The supernatants were removed and 200 μl DMSO was added to the wells, and the plates were shaken for 10 min. The absorbance was measured at 540 nm by a plate reader (Synergy HT, BioTek).

### Apoptotic Cell detection by EB/AO Staining

Cells were cultured in 96-well plates at a confluence of 1 × 10^3^ cells/well, incubated overnight and then treated with compounds in their 24 h specific IC_50_ doses. Then plates were centrifuged for 5 min (129 *g*, 1,000 rpm) at 4 °C. The ethidium bromide/acridine orange (EB/AO) dye mix (100 μg/mL ethidium bromide and 100 μg/mL acridine orange) was dissolved in PBS and 20 μl of the dye mix was added to wells. Cells were counted by an inverted fluorescence microscope (IX 71, OLYMPUS). Live cells were determined by normal green color, which resulted from up-taking acridine orange (green fluorescence) and repelling ethidium bromide (red fluorescence).

Apoptotic cells display apoptotic bodies and perinuclear condensation of chromatin stained by ethidium bromide, while live cells were identified as normal nuclear chromatin stained by acridine orange. Necrotic cells were detected by uniform ethidium bromide staining of the cells^7^. Images were captured with a digital camera (DP 71, OLYMPUS) equipped microscope. Experiments were done in triplicate, quantifying a minimum of 100 cells each time.

### Flow Cytometric analysis of the Cell Cycle

Cells were cultured in 6-well plates at a confluence of 1 × 10^6^ cells/well. Cells were treated with compounds for 24 h with respective IC_50_ values. Then, cell cycle phases and DNA content were analyzed by flow cytometry. Briefly, cells were collected and fixed with ice ethanol 70% for 2 h. Fixed cells were centrifuged (300 *g*, 4 °C, 5 min) and washed with cold PBS, and then stained with diamidino-2-phenylindole (DAPI, 10 μg/mL, Triton X-100 0.1% v/v in PBS). Then, cells were filtered by a nylon mesh with a pore size of 30 μm. Cell cycle analysis was done using a flow cytometer (Partec CyFlow space, Germany). The distribution of cells in different cell cycle phases was assessed by Partec FloMax software.

### mRNA expression analysis by qPCR

Cells were cultured in 6-well plates at a confluence of 2 × 10^5^ cells/well and kept at 37 °C in a moistened air of 5% CO2 overnight. Then, cells were treated for 48 h with respective IC_50_ values.

For RNA extraction from cells, Trizol reagent (Cat. No: 15596-026, Invitrogen, CA, USA) was used according to the manufacturer’s protocol. First-strand cDNA was generated from the cells’ extracted RNA by the RevertAid First Strand cDNA Synthesis Kit, Fermentas (Cat No: #K1621, Maryland, USA) according to manufacturer’s directions.

Primers (Bax, cyclin D1, VEGFA, Bcl-2, Caspase 3, c-myc, survivin and the *Homo sapiens* ribosomal protein L38 (RPL38) as a housekeeping gene) were designed using Primer Express 3.0 (PE Applied Biosystems, Foster City, CA, USA). See Table 2 for the details of primers used in quantitative real-time PCR. For accuracy and specificity, all primers were blasted in the NCBI website: http://www.ncbi.nlm.nih.gov/tools/primer-blast/. Primers were synthesized by the custom oligonucleotide synthesis service, Metabion (Martinsried, Germany).

**Table 2:** Primers used in quantitative PCR and the amplicon sizes (bp: base pair).

Target	Forward primer	Reverse primer	Amplicon Size (bp)
Bax	5′-GCCCTTTTGCTTCAGGGTTTC	5′-CATCCTCTGCAGCTCCATGT	168
Cyclin D1	5′-GGCGGAGGAGAACAAACAGA	5′-TGTGAGGCGGTAGTAGGACA	181
VEGFA	5′-TGTCTAATGCCCTGGAGCCT	5′-GCTTGTCACATCTGCAAGTACG	175
Bcl-2	5′-CAGGATAACGGAGGCTGGGATG	5′-AGAAATCAAACAGAGGCCGCA	70
Caspase 3	5′-GCGGTTGTAGAAGAGTTTCGTG	5′-CTCACGGCCTGGGATTTCAA	101
c-myc	5′-CCCTCCACTCGGAAGGACTA	5′-GCTGGTGCATTTTCGGTTGT	96
Survivin	5′-TTCAAGGAGCTGGAAGGCTG	5′-AGCAATGAGGGTGGAAAGCA	151
RPL38	5′-TCACTGACAAAGAGAAGGCAGAG	5′-TCAGTGTGTCTGGTTCATTTCAGTT	88

Quantitative analysis was done using StepOne Real-Time PCR System (Applied Biosystems 7500, Foster City, CA, USA) with the PowerSYBR Green PCR Master Mix (Cat. No: 4309155, Applied Biosystems, Foster City, CA, USA).

Individual reaction mix contained an overall volume of 25 μl (master mix 12.5 μl, cDNA 3 μl, primer 3 μl, and H2O 6.5 μl). Thermocycling conditions were as follows: 50 °C for 2 minutes, 95 °C for 10 minutes, then 40 cycles of 95 °C for 30 seconds, 60 °C 30 seconds, and 72 °C for 30 seconds.

Relative quantities of target mRNA in test samples were measured and standardized to the housekeeping gene, RPL38 mRNA transcript level. The comparative Ct method was used to assess expression as previously described by Livak and Schmittgen^8^.

### Statistical Analysis

IC_50_ values were analyzed using Sigma Plot 12 software. Values for the growth inhibition study are presented as mean ± SD, except in figures where error bars represent standard error of mean (SEM).

## Results

**Figure 3:**
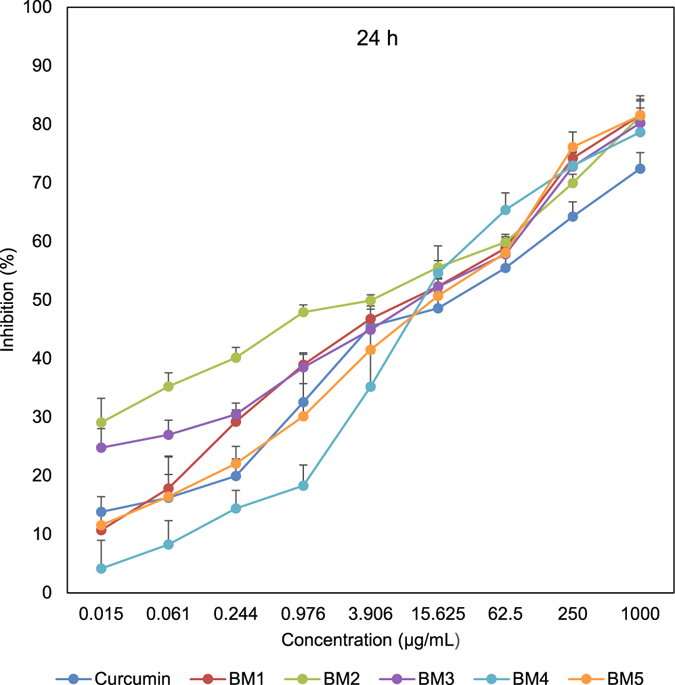
Inhibitory effect of synthesized compounds on AGS cells assessed with MTT assay at 24 h time point.

**Figure 4:**
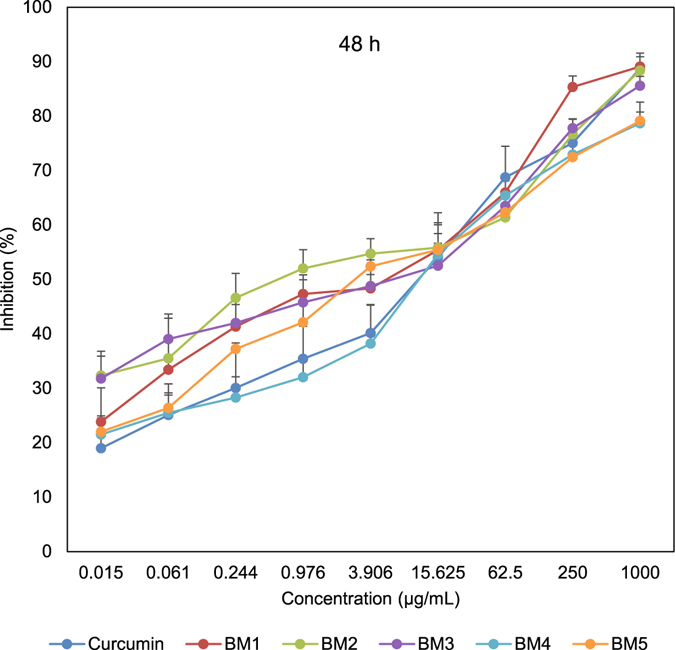
Inhibitory effect of synthesized compounds on AGS cells assessed with MTT assay after 48 h time point.

**Figure 5:**
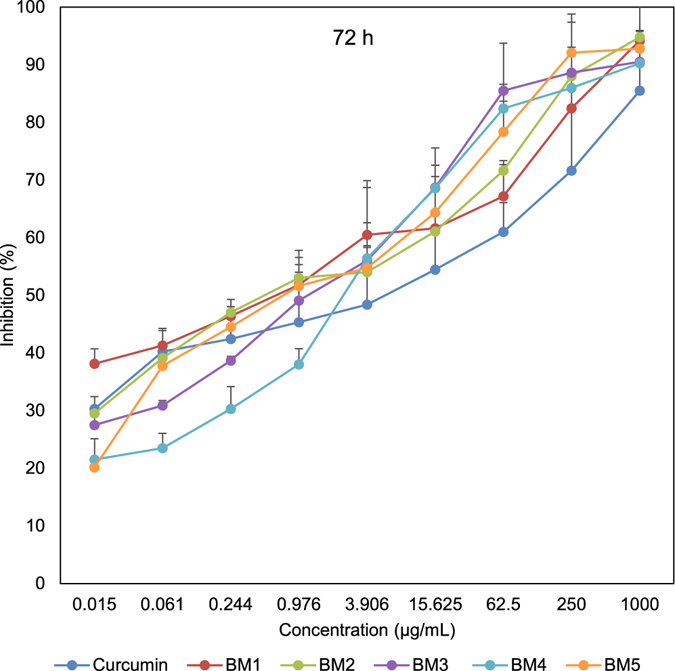
Inhibitory effect of synthesized analogues on AGS cells determined by MTT assay after 72 h time point.

In order to assess the effect of synthesized compounds on cell proliferation, an MTT assay was conducted to test the inhibitory effect in three time points. After 24 h, all generated analogues were cytotoxic toward gastric and esophageal cancer cells and showed lower IC_50_ values than curcumin. As shown in Table 3 and Fig. 3, BM2 was 4.6 times more toxic than curcumin toward gastric cancer cells. Similarly, esophageal cancer cells were more susceptible to BM2 and other synthesized compounds than curcumin (Supplementary Table S1). We observed the same pattern after 48 h, with BM2 17 times more toxic than curcumin (Table 3, and Fig. 4). Similarly, 72 h post treatment, all compounds were more effective than curcumin. Three curcumin analogues revealed IC_50_ with Nanogram/mL values (Table 3, and Fig. 5). Moreover, MTT assay on KYSE-30 cells confirmed our data and showed that synthesized compounds have cytotoxicity on esophageal cancer cells as well (Supplementary Table S1 and Fig. S1). These data revealed that all synthesized analogues showed IC_50_ much less than curcumin in three time points.

**Table 3:** IC50 values of synthetized 2,6-Bis Benzylidine cyclohexanone analogues in AGS cells that analyzed by MTT assay after 24 h, 48 h, and 72 h. Values are in µg/mL.

Analogue	24 h	48 h	72 h
Curcumin	18.43	12.33	5.42
BM1	9.85	5.45	0.72
BM2	3.95	0.72	0.56
BM3	11.71	7.53	1.39
BM4	14.15	4.35	1.96
BM5	15.22	3.72	0.83

**Figure 6:**
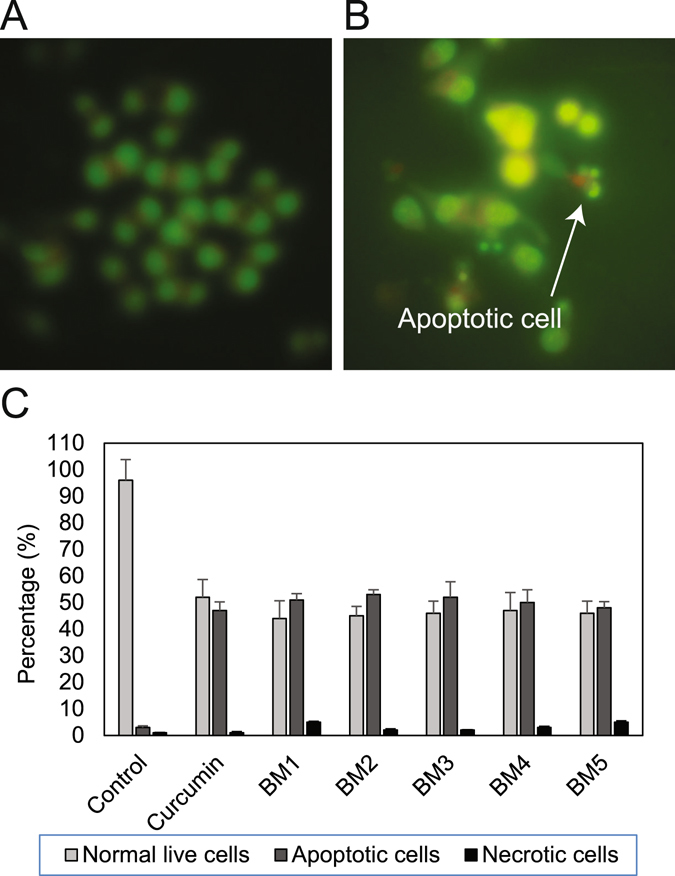
EB/AO staining for detecting apoptotic cells. (**A**) Control un-treated AGS cells. (**B**) Representative micrograph of apoptotic cells treated with BM2 that are characterized with fragmented and condensed nuclei of cells. (**C**) Quantification of the cells with normal, necrotic and apoptotic representation.

In order to elucidate the mode of cell death, cells were stained with EB/AO, and apoptotic, necrotic, and live cells were counted. Synthetic compounds changed the morphology of treated cells to characteristic apoptotic cells. Nuclei of treated cells condensed and revealed fragmented chromatin and apoptotic bodies. As presented in Fig. 6B, treatment of AGS cells with synthesized BM2 triggered apoptotic cell death, which is characterized by fragmentation of nuclei. Quantification of treated and control cells revealed that synthesized analogues increased the number of apoptotic cells significantly compared to control cells (Fig. 6C and Fig. S2).

**Figure 7:**
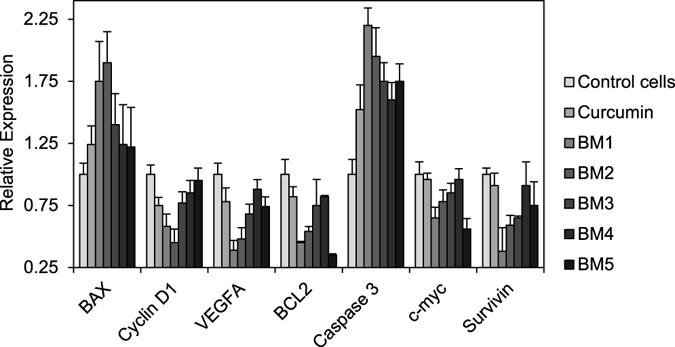
Relative expression levels of Bax, cyclin D1, VEGFA, Bcl-2, Caspase 3, c-myc, survivin and RPL38 mRNA in AGS cells. All Ct values were normalized with the *homo sapiens* ribosomal protein L38 (RPL38) as a housekeeping gene.

In order to verify that compounds trigger the apoptosis pathway, mRNA expression levels of important apoptotic factors were analyzed. Treatment with synthesized compounds elevated BAX and caspase-3 mRNA levels, and down-regulated expression of cyclin-D1, VEGFA, Bcl-2, c-myc, and Survivin (Fig. 7 and Fig. S3).

**Figure 8:**
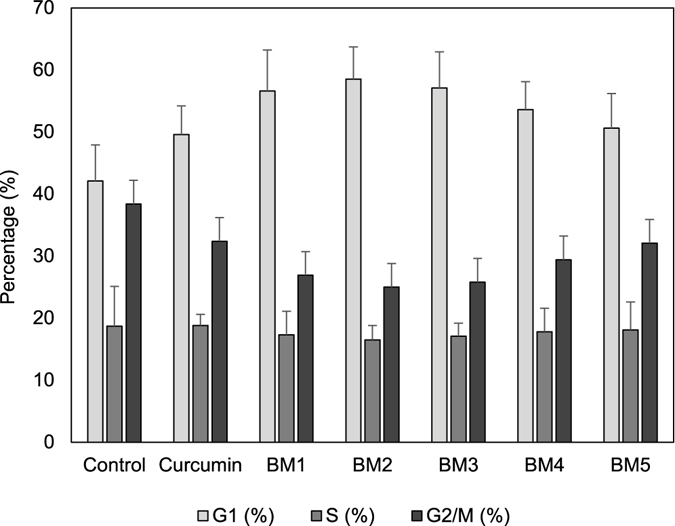
Flow cytometric analysis of the cell cycle. Curcumin analogues altered distribution of the AGS cells in cell cycle phases, which indicates that cell cycle arrested at G1 phase.

In order to further confirm anti-proliferative effects, cell cycle distribution of the treated cells was evaluated. As displayed in Fig. 6, synthesized compounds were more potent than curcumin at arresting the cell cycle at G1 phase. BM2 and BM3 were most effective at increasing the cell population at G1 phase. Simultaneously, analogues decreased the number of cells at S phase (Fig. 8 and Fig. S4).

## Discussion

To date over 100 different clinical trials have been completed with curcumin which clearly shows its safety, tolerability, and effect against numerous chronic diseases in humans^3^.

Curcumin, a polyphenolic natural product, shows therapeutic function against a variety of diseases. These activities are attributed mainly to its chemical structure and unique physical, chemical, and biological properties. It is a di-feruloyl methane molecule [1,7-bis (4-hydroxy-3- methoxyphenyl)-1,6-heptadiene-3,5-dione)] containing two ferulic acid residues linked by a methylene connection. It has three key functional groups: an aromatic o-methoxy phenolic group, α, β-unsaturated β-diketo moiety and a seven carbon linker^9^. In this study we designed different analogues of curcumin with non-polar and hydrophobic groups (methoxy, propoxy, ethoxy, and allyloxy), in order to test the effect of less polar synthesized analogues on cancer cell proliferation and compare them with the anti-cancer effect of curcumin.

Recently, it has been reported that replacement of two OH groups in curcumin with less polar groups like methoxy (OCH_3_) increases the anti-proliferative activity of arene-ruthenium(II) curcuminoid complexes in tumor cells. This report states that improved anti-cancer function is associated with apoptotic activity^10^. These findings are in accordance with our results: by decreasing curcumin polarity, its *in vitro* anti-tumor activity increases significantly. Our various experimental results on esophageal and gastric cancer cells revealed that all synthesized analogues of curcumin are more toxic than curcumin: after 48 h treatment in gastric cancer cells, BM2 was 17 times more toxic than curcumin (Fig. 4).

Very recently it is reported that curcumin triggered cell cycle arrest at G1 phase, and reduced the cell population in S phase in p53-mutated human colon adenocarcinoma cells^11^. These finding are in agreement with our data, which demonstrate that synthesized analogues increase cell population at G1 phase and decrease cell population at S phase (Fig. 8).

The same study reports that curcumin induced apoptosis in p53-mutated COLO 320DM colon adenocarcinoma cells^11^. Our novel synthesized compounds were more effective than curcumin at triggering apoptosis (Figs 6 and 7 and Supplementary Figs S2–S4).

It has been reported that curcumin induced apoptosis in leukemia cells by PARP-1 cleavage, increased level of caspase-3, apoptosis inducing factor (AIF) and down-regulation of Bcl2^12^. Another study suggested that dendrosomal curcumin (DNC) significantly increased cell population in SubG1, induced apoptosis and up-regulated p21, BAX, and Noxa in hepatocarcinoma cell lines. While the expression of Bcl-2 decreased^13^. Moreover, it has been reported that a curcumin analogue ((1E, 6E)-1, 7-di (1H-indol-3-yl) hepta-1, 6-diene-3, 5-dione) down-regulated cyclin D1 and activated Caspase 3, 8 and 9 in lung adenocarcinoma (A549), leukemia (K562) and colon cancer (SW480) cells^14^. Another study showed that curcumin suppressed VEGF secretion from tumor cells both *in vitro* and *in vivo*, and subsequently could block VEGF-VEGFR2 signaling pathways^15^. It has been evidenced that curcumin combination with resveratrol synergistically induced apoptosis in cigarette smoke condensate transformed breast epithelial cells by increasing Bax/Bcl-xL ratio, Cytochrome C release, cleaved product of PARP and caspase 3. Whereas, this combination decreased c-myc and cyclin-D1^16^. Nevertheless, it has been demonstrated that dimethoxy curcumin (DMC) as a non-polar and lipophilic analogue of curcumin down-regulating survivin and upregulating E-cadherin in colon cancer cells which significantly suppressed the growth and migration of cells^17^.

Our novel synthesized compounds potentially up-regulated Bax and caspase-3 and down-regulated anti-apoptotic players (Fig. 7).

Cyclin D1 is one of the G1 phase related regulatory factors^18^; its down-regulation in our results verifies potential effect of novel analogues on cell cycle arrest at G1 phase (Fig. 8).

Taken together, novel synthetic 2,6-bis benzylidine cyclohexanone analogues were more efficient than curcumin to inhibit cancer cell proliferation, trigger apoptosis, and arrest cell cycle at G1 phase. These data suggest that cyclohexanone analogues of curcumin could be promising anti-cancer agents to consider for more research on animal tumor models and even human clinical trials.

## Electronic supplementary material

Supplementary Information.pdf
