# Supplementary material for: Potent anti-cancer effects of less polar Curcumin analogues on gastric adenocarcinoma and esophageal squamous cell carcinoma cells
Source: Sci Rep. 2017 May 31;7:2559. doi: 10.1038/s41598-017-02666-4 (PMC5451386; doi:10.1038/s41598-017-02666-4)
Supplement: Supplementary file 1 — Supplementary Information.pdf [file 41598_2017_2666_MOESM1_ESM.docx]

**Supplementary Information**

**Potent anti-cancer effects of less polar Curcumin analogues on gastric adenocarcinoma and esophageal squamous cell carcinoma cells**

Fatemeh Alibeiki, Naser Jafari, Maryam Karimi , Hadi Peeri Dogaheh

**Supplementary Table S1:** IC50 values of synthetized 2,6-Bis Benzylidine cyclohexanone derivatives on KYSE-30 cells that analyzed by MTT assay after 24h, 48h, and 72h time points. Values are in µg/ml.

| Extract | 24h | 48h | 72h |
| --- | --- | --- | --- |
| Curcumin | 246.7 | 174.7 | 15.3 |
| BM1 | 118.4 | 53.2 | 3.6 |
| BM2 | 78.3 | 46.8 | 3.4 |
| BM3 | 158.2 | 58.7 | 9.7 |
| BM4 | 155.5 | 81.3 | 12.7 |
| BM5 | 231.4 | 86.1 | 14.1 |

**Supplementary Figure S1:** Inhibitory effect of synthesized compounds at KYSE-30 cells assessed with MTT assay at 24, 48 and 72 h time point.


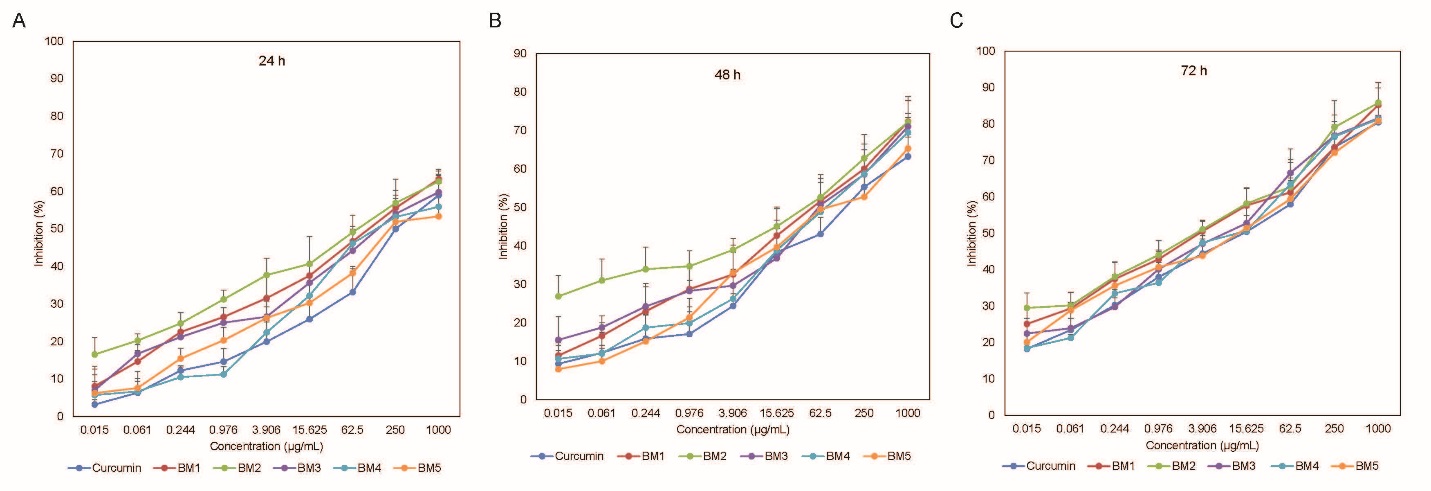


**Supplementary Figure S2:** 2,6-Bis Benzylidine cyclohexanone derivatives induced apoptosis in the KYSE-30 cells. Quantification of the KYSE-30 cells with normal, necrotic and apoptotic representation that acquired using EB/AO staining.

**Supplementary Figure S3**: 2,6-Bis Benzylidine cyclohexanone derivatives dys-regulated expression levels of Bax, cyclin D1, VEGFA, Bcl-2, Caspase 3, c-myc and survivin in the KYSE-30 cells. All Ct values were normalized with the *homo sapiens* ribosomal protein L38 (RPL38) as a housekeeping gene.

**Supplementary Figure S4:** 2,6-Bis Benzylidine cyclohexanone derivatives arrested cell cycle at G1 phase in the KYSE-30 cells.
